# The Effect of Selected Synbiotics on Microbial Composition and Short-Chain Fatty Acid Production in a Model System of the Human Colon

**DOI:** 10.1371/journal.pone.0047212

**Published:** 2012-10-17

**Authors:** Gabriella C. van Zanten, Anne Knudsen, Henna Röytiö, Sofia Forssten, Mark Lawther, Andreas Blennow, Sampo J. Lahtinen, Mogens Jakobsen, Birte Svensson, Lene Jespersen

**Affiliations:** 1 Department of Food Science, Faculty of Life Sciences, University of Copenhagen, Frederiksberg, Denmark; 2 Enzyme and Protein Chemistry, Department of Systems Biology, Technical University of Denmark, Kgs. Lyngby, Denmark; 3 Department of Plant Biology and Biotechnology, Faculty of Life Sciences, University of Copenhagen Frederiksberg, Denmark; 4 DuPont Nutrition and Health, Kantvik, Finland; 5 Biovelop A/S, Kvistgård, Denmark; Charité-University Medicine Berlin, Germany

## Abstract

**Background:**

Prebiotics, probiotics and synbiotics can be used to modulate both the composition and activity of the gut microbiota and thereby potentially affecting host health beneficially. The aim of this study was to investigate the effects of eight synbiotic combinations on the composition and activity of human fecal microbiota using a four-stage semicontinuous model system of the human colon.

**Methods and Findings:**

Carbohydrates were selected by their ability to enhance growth of the probiotic bacteria *Lactobacillus acidophilus* NCFM (NCFM) and *Bifidobacterium animalis* subsp. *lactis* Bl-04 (Bl-04) under laboratory conditions. The most effective carbohydrates for each probiotic were further investigated, using the colonic model, for the ability to support growth of the probiotic bacteria, influence the composition of the microbiota and stimulate formation of short-chain fatty acids (SCFA).The following combinations were studied: NCFM with isomaltulose, cellobiose, raffinose and an oat β-glucan hydrolysate (OBGH) and Bl-04 with melibiose, xylobiose, raffinose and maltotriose. All carbohydrates showed capable of increasing levels of NCFM and Bl-04 during fermentations in the colonic model by 10^3^–10^4^ fold and 10–10^2^ fold, respectively. Also the synbiotic combinations decreased the modified ratio of *Bacteroidetes/Firmicutes* (calculated using qPCR results for *Bacteroides-Prevotella-Porphyromonas* group, *Clostridium perfringens* cluster I, *Clostridium coccoides - Eubacterium rectale* group and Clostridial cluster XIV) as well as significantly increasing SCFA levels, especially acetic and butyric acid, by three to eight fold, as compared to the controls. The decreases in the modified ratio of *Bacteroidetes*/*Firmicutes* were found to be correlated to increases in acetic and butyric acid (p = 0.04 and p = 0.03, respectively).

**Conclusions:**

The results of this study show that all synbiotic combinations investigated are able to shift the predominant bacteria and the production of SCFA of fecal microbiota in a model system of the human colon, thereby potentially being able to manipulate the microbiota in a way connected to human health.

## Introduction

The concept of prebiotics was introduced by Gibson & Roberfroid [1]. The vast majority of published studies have focused on the effects of inulin, fructo-oligosaccharides and galacto-oligosaccharides [2]. Prebiotics have been reported to selectively stimulate growth of bifidobacteria, and to lesser extent lactobacilli, both *in vitro* and in human trials [3]–[5]. Human trials have indicated stimulation of mineral uptake [6], [7] and an influence on cholesterol levels by reduction of triacylglycerol concentrations in blood by prebiotics [8], and animal studies suggest an effect of prebiotics on reduction of cancer risk [9], [10]. Abrams *et al.* (2007) reported prebiotics to have an effect on BMI of adolescents [11]. Two studies have assessed the effect of prebiotics on irritable bowel syndrome and reported reduced frequency and severity of abdominal pain [12] and reduction of flatulence, bloating, abdominal pain together with self-reported global assessment of relief [13]. As reviewed by Roberfroid *et al*. [2], several pilot studies suggest an effect of prebiotics on inflammatory bowel disease (*i.e.* on disease activity), which is supported by a larger trial reporting reduction of inflammation in pouch mucosa in patients with pouchitis [14]. There is however also a risk of side effects due to prebiotic intake as reported by a study with patients with active Crohn’s disease, where gastrointestinal symptoms (*i.e*. flatulence and abdominal pain) were increased [15]. Also, no difference in disease activity and markers of intestinal inflammation was reported, however, expression of IL-10 and IL-6 by intestinal dendritic cells were shifted in a anti-inflammatory manner [15].

For probiotic bacteria, beneficial health effects have been claimed since Metchnikoff, and have been reported in numerous *in vitro* studies as reviewed recently [16]. Beneficial effects have also been shown in human intervention studies addressing e.g. the effect of a mixture of probiotic *Lactobacillus rhamnosus* and *Lactobacillus reuteri* on atopic dermatitis [17] and diarrhea [18]. Probiotic strains *L. rhamnosus* GG and *L. rhamnosus* HN001 have been reported to reduce prevalence of eczema [19], [20]. Moreover the probiotic strain *Bifidobacterium animalis* subsp. *lactis* Bi-07 has been reported to reduce the severity of atopic dermatitis as compared to before probiotic treatment, however no difference was observed between probiotic and placebo treatment [21]. Perinatal exposure to *L. rhamnosus* GG showed a tendency of restraining excessive weight gain during the first years of life [22] and *L. acidophilus* NCFM preserved insulin sensitivity in type 2 diabetics as compared to placebo, however inflammatory markers and systemic inflammatory response were unaffected [23].

Combining probiotic bacteria with prebiotics, i.e. so-called synbiotics, to gain the health beneficial effects of both has been suggested, and has been investigated both *in vitro* and in clinical trials. The beneficial effects reported for humans include favorable shift in cancer biomarkers in colon cancer patients [24] and improvement of liver function in cirrhotic patients [25]. A study reported post-natal synbiotic treatment of infants to result in lower frequency of antibiotic treatment during trial and respiratory infections occurred less frequent during the follow-up period [26]. A study of the effect of synbiotics on post-operative infections reported a lower bacterial infection rate as well as shorter duration time of antibiotic treatment as compared to placebo [27]. Also, certain synbiotics have been observed to increase levels of lactobacilli and bifidobacteria [24], [28].

It is generally accepted that increases in lactobacilli and bifidobacteria are desirable, and most known probiotics belong to these genera [29]. However, it has been estimated that the colon contains 500 to 1000 different bacterial species, which may be linked to the health status of the host [30]. Recently particular interest has been given to altered ratios of *Bacteroidetes/Firmicutes*. Ratios are reported to be altered in obese [31], [32] and type 2 diabetics [33]. However, other studies report no changes in *Bacteroidetes/Firmicutes* ratio in obese as compared to lean subjects [34], [35]. Metabolic activity of the gut microbiota results in production of short-chain fatty acids (SCFA), mainly acetic, propionic and butyric acids which serve as fuel for the intestinal epithelial cells and stimulate growth of colonic epithelial cells [36], [37]. Butyric acid and propionic acid are reported to inhibit growth and promote apoptosis of human colonic carcinoma cell lines [38], [39], while anti-inflammatory properties have been reported for acetic, propionic and butyric acid [40]. It was recently demonstrated that acetic acid produced by bifidobacteria stimulate epithelial cell defense against infection by *Escherichia coli* O157:H7 [41].

Studying the complex microbial community of the human colon presents methodological challenges but despite limitations, colonic models are seen as useful tools for *in vitro* investigation of the composition and metabolism of colonic bacteria [42]. The models have been used to study the interaction of the opportunistic pathogen *Staphylococcus aureus* and colonic microbial population [43], survival of probiotics [44] and have been used to investigate effects of known prebiotics on the microbial community [45] as well as for identification of novel prebiotic candidates [46]. The four-stage colonic model used in this study has been validated by human trials with regard the bifidogenic effect of a synbiotic combination of NCFM and lactitol and enhancement of SCFA by polydextrose [3], [47]–[50].

In the present study 37 potentially prebiotic carbohydrates were investigated for their ability to enhance the growth of the widely used and well studied probiotic bacteria *Lactobacillus acidophilus* NCFM and *Bifidobacterium animalis* subsp. *lactis* Bl-04 [23], [51]–[55]. Eight synbiotic combinations were selected for further analysis in the colonic model; NCFM combined with isomaltulose, cellobiose, raffinose or endo-1,3-β-D-glucanase hydrolyzed oat β-glucan, and Bl-04 combined with melibiose, xylobiose, raffinose or maltotriose. Performance in the four-stage model of the human colon inoculated with human fecal samples was evaluated with respect to growth stimulation of the probiotic strains by qPCR and production of short-chain fatty acids, investigated by gas chromatography. Moreover, to obtain quantitative information of the effects of synbiotics on the modified ratio of *Bacteroidetes/Firmicutes,* qPCR was applied for determination of microbial numbers.

## Results

### Selection of Potential Prebiotic Carbohydrates for Stimulation of Growth of *L. acidophilus* NCFM and *B. animalis* subsp. *lactis* Bl-04

A library of potentially prebiotic carbohydrates (Table S1) was investigated for the growth enhancement of NCFM and Bl-04 (Figure S1). Although distinct differences were observed between NCFM and Bl-04, both preferred carbohydrates with a DP between two to five. Apart from glucose (control) and galactose (no. 6) which stimulated growth of NCFM, the monosaccharides included in this study did not result in growth enhancement of neither NCFM nor Bl-04. Disaccharides such as isomaltose (no. 10) and gentiobiose (no. 12) stimulated growth of both NCFM and Bl-04 while cellobiose (no. 11) strongly enhanced growth of NCFM and to some extent Bl-04. Melibiose (no. 8) and xylobiose (no. 17) only stimulated growth of Bl-04 and isomaltulose (no. 9) strongly enhanced growth of NCFM but not Bl-04. Among the trisaccharides D-raffinose (no. 18) stimulated growth of both NCFM and Bl-04, panose (no. 19) stimulated growth of Bl-04, and to some extent NCFM, while maltotriose (no. 20) highly stimulated growth of Bl-04 but not NCFM. The tetrasaccharide stachyose (no. 21) and the pentasaccharide verbascose (no. 22) stimulated growth of both NCFM and Bl-04. No, or very limited, *in vitro* growth enhancement was observed for carbohydrates with DP above five. Based upon the screening the following combinations of carbohydrates and probiotic bacteria were selected for studies in the colonic model. Being able to support growth of both NCFM and Bl-04, raffinose (no.18), in combinations with both bacteria, isomaltulose (no. 9) and cellobiose (no. 11) in combination with NCFM and melibiose (no. 8), xylobiose (no. 17) and maltotriose (no. 20) in combination with Bl-04 were selected for evaluation in the colonic model of the human colon. Oat β-glucan has previously been reported to increase cecal numbers of *L. acidophilus* [56], therefore oat β-glucan hydrolyzed (OBGH) by endo-1,3-β-D-glucanase (no. 26) was included in combination with NCFM although no growth enhancement was observed under laboratory conditions.

### Effects of Selected Carbohydrates on Growth Enhancement of *L. acidophilus* NCFM, *B. animalis* subsp *lactis* Bl-04 in the Colonic Model System

In fermentations with NCFM, all combinations increased NCFM numbers in the order of 10^2^–10^4^ fold (Table 1A) with the levels of NCFM slightly decreasing throughout the colonic model.

**Table 1:** *Lactobacillus acidophilus* NCFM and *Bifidobacterium animalis* subsp. *lactis* Bl-04 numbers (log_10_ bacteria/mL ± SE) detected by quantitative PCR for colonic fermentations with A) NCFM and carbohydrates stated and B) Bl-04 and carbohydrates stated (n = 2 for all except Bl-04 and maltotriose and control fermentations where n = 3).

A		control	NCFM+isomaltulose	NCFM+ cellobiose	NCFM+ raffinose	NCFM+ OBGH^1^
*L. acidophilus* NCFM	V1	4.14 (±0.65)	7.65 (±0.21)*	7.57 (±0.01)*	7.58 (±0.28)*	7.51 (±0.19)*
	V2	4.19 (±0.32)	7.10 (±0.03)*	7.52 (±0.01)**	7.37 (±0.46)*	6.94 (±0.29)*
	V3	3.23 (±0.33)	6.70 (±0.08)**	7.21 (±0.06)**	7.01 (±0.25)**	6.19 (±0.30)*
	V4	3.06 (±0.16)	6.38 (±0.19)**	6.65 (±0.05)**	6.63 (±0.31)**	5.01 (±0.64)*
**B**		**control**	**Bl-04+ melibiose**	**Bl-04+ xylobiose**	**Bl-04+ raffinose**	**Bl-04+ maltotriose** 2
*B. animalis* subsp. *lactis* Bl-04		4.84 (±0.22)	6.25 (±0.35)*	6.30 (±0.50)	7.10 (±0.14)*	6.91 (±0.33)*
		4.76 (±0.27)	6.01 (±0.24)*	6.48 (±0.45)*	7.18 (±0.15)*	7.13 (±0.30)**
		4.39 (±0.32)	5.44 (±0.28)	5.92 (±0.49)	6.48 (±0.21)*	6.70 (±0.28)**
		4.25 (±0.39)	4.96 (±0.48)	5.46 (±0.56)	5.92 (±0.21)*	6.08 (±0.02)*

* p<0.05.

** p<0.005.

1 Oat β-glucan hydrolysate.

2 Maltotriose from Sigma-Aldrich.

For the fermentations with Bl-04 and the selected carbohydrates resulted in an increase of Bl-04 in the order of 10^2^ for the combinations with raffinose and maltotriose, and in the order of 10 for the combinations with melibiose and xylobiose (Table 1B). Also for the synbiotic combinations with Bl-04, the numbers decreased slightly throughout the colonic model.

For NCFM in combination with isomaltulose or cellobiose, numbers of bifidobacteria were increased by a factor 10 and NCFM in combination with raffinose increased bifidobacteria by a factor 10^2^ (data not shown). The combination of NCFM and OBGH did not affect levels of bifidobacteria. The fermentations of Bl-04 and raffinose interestingly, decreased levels of lactobacilli, in the order of 10 fold, in vessels V1–V2 while the combination of Bl-04 with melibiose decreased levels of lactobacilli in the order of 10–10^3^ fold (data not shown). The combinations of Bl-04 and xylobiose or maltotriose increased lactobacilli numbers in the range of a factor 10–10^2^.

Numbers of *Lactobacillus* spp. and *Bifidobacterium* spp. were also determined, however numbers of NCFM and Bl-04 were higher than that of lactobacilli and bifidobacteria, respectively. This was however not observed for control simulations, indicating a PCR-bias due to high levels of NCFM and Bl-04, respectively, and these results have therefore not been included.

### Effects of Selected Combinations of Carbohydrates, *L. acidophilus* NCFM and *B. animalis* subsp. *lactis* Bl-04 on the Composition of the Colonic Model System Microbiota

**Figure 1:**
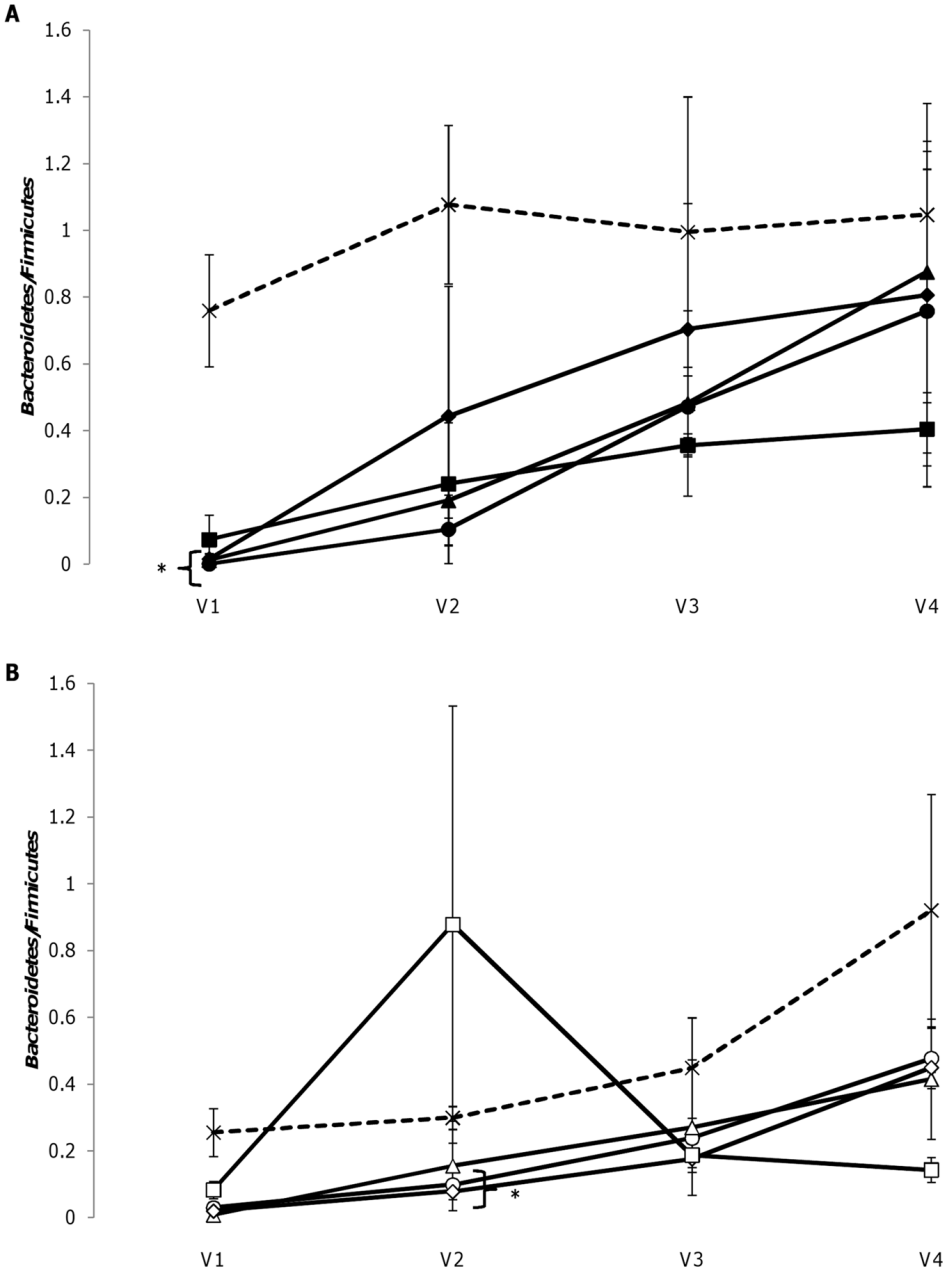
Ratios of *Bacteroidetes/Firmicutes* as determined by qPCR. Ratios of *Bacteroidetes/Firmicutes* (*Bacteroides-Prevotella-Porphyromonas* group/*Clostridium perfringens* cluster I, *Clostridium coccoides - Eubacterium rectale* group and Clostridial cluster XIV) for fermentations with *Lactobacillus acidophilus* NCFM (A) in combination with isomaltulose (•) (n = 2), cellobiose (▴)(n = 2), raffinose (♦)(n = 2) and OBGH (▪)(n = 2); *Bifidobacterium animalis* subsp. *lactis* Bl-04 (B) in combination with melibiose (**○**)(n = 2), xylobiose (Δ) (n = 2), raffinose (◊)(n = 2) and maltotriose (□)(n = 3). Control fermentations (n = 3) are denoted by crosses and dotted lines and results are shown as mean values for each vessel, V1–V4, ± standard error of mean. *p<0.05.

Numbers of *Enterobacteriaceae, Bacteroides-Prevotella-Porphyromonas* group belonging to *Bacteroidetes* and *Faecalibacterium prausnitzii, Clostridium perfringens* cluster I, *Clostridium coccoides* - *Eubacterium rectale* group and Clostridial cluster XIV belonging to the *Firmicutes*, were assessed by qPCR, To avoid a complex description of the changes the modified ratio *Bacteroidetes*/*Firmicutes* was used to describe shifts in the microbial composition and the modified ratio showed a tendency of being decreased by all synbiotic combinations as seen in Figure 1. (*Firmicutes* was calculated using numbers of *Clostridium perfringens* cluster I, *Clostridium coccoides - Eubacterium rectale* group and Clostridial cluster XIV). Numbers of *F. prausnitzii* were constant for all synbiotic combinations and were 10 fold higher than the sum of the other bacteria, likely due to PCR bias, and *F. prausnitzii* was therefore not included in calculations of the modified ratio. For the combination of Bl-04 and maltotriose an outlier in V2 was not included. The decrease in the modified ratio was most pronounced in the first and second vessels of the model system. For fermentations of NCFM synbiotics, all combinations decreased modified *Bacteroidetes*/*Firmicutes* ratio in vessel V1 (p>0.05) except of the combination of NCFM with OBGH (p = 0.05). All fermentations showed tendency to decrease the ratio in V2, especially NCFM in combination with isomaltulose, cellobiose and OBGH (p = 0.05 to 0.09). In the fermentations with Bl-04, ratio showed tendency of decrease for all combinations, in particular combinations of Bl-04 with melibiose, xylobiose and raffinose in V1 (p = 0.08 to 0.09). Ratios in V2, of the colonic model were lower for Bl-04 in combination with melibiose or raffinose (p<0.05).

Average levels of *Enterobacteriaceae* were in the range of 4,6–5.0 (log_10_ bacteria/mL) and were not changed by the synbiotic combinations (data not shown).

### Effects of Selected Carbohydrates, *L. acidophilus* NCFM and *B. animalis* subsp. *lactis* Bl-04 on Production of Volatile Fatty Acids in the Colonic Model System

**Figure 2:**
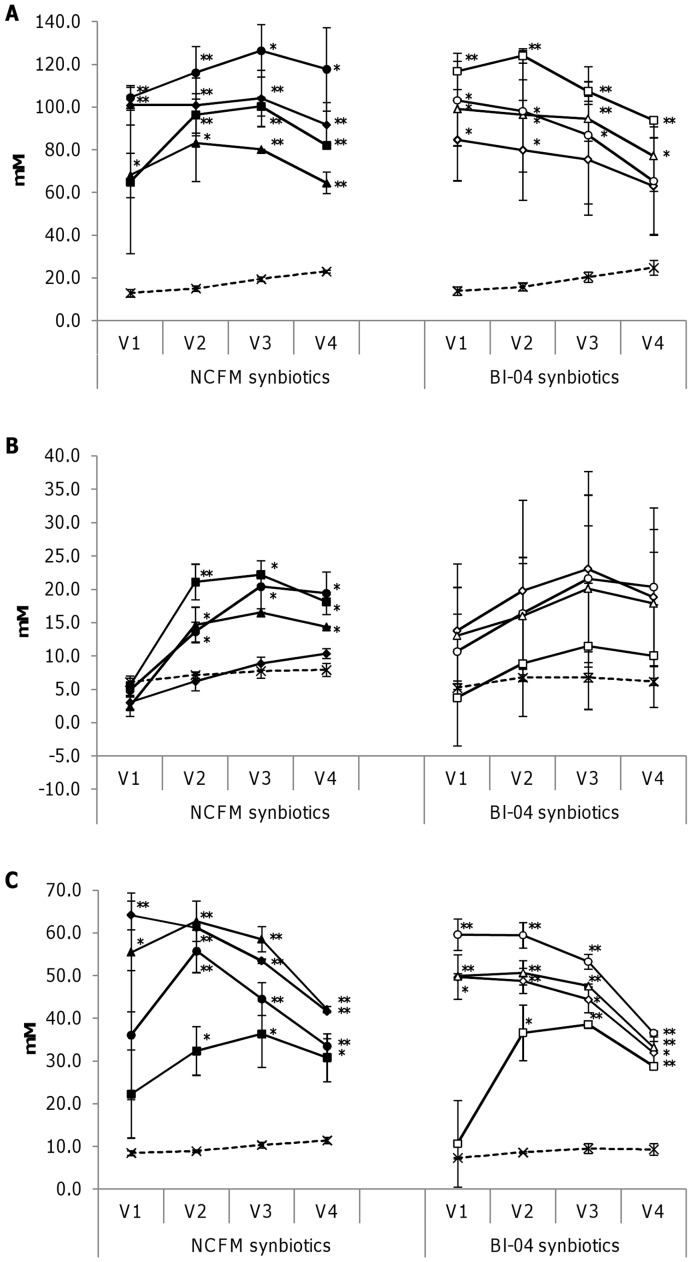
Concentrations of short-chain fatty acids as determined by gas chromatography; acetic acid (A), propionic acid (B) and butyric acid (C) in vessels V1–V4 of the colonic model after control and synbiotic fermentations. Synbiotic fermentations are denoted as follows: *Lactobacillus acidophilus* NCFM in combination with; isomaltulose (•)(n = 2), cellobiose (▴)(n = 2), raffinose (♦)(n = 2) and OBGH (▪)(n = 2) and *Bifidobacterium animalis* subsp. *lactis* Bl-04 in combination with; melibiose (○)(n = 2), xylobiose (Δ) (n = 2), raffinose (◊)(n = 2) and maltotriose (□)(n = 3). Control fermentations (n = 3) are denoted by crosses and dotted lines and results are shown as mean concentrations (mmol/L) for each vessel ± standard error of mean. *p<0.05, **p<0.005.

The concentrations of short-chain fatty acids (SCFA) and branched-chain fatty acids (BCFA) produced during the fermentations in the colonic model are presented in Figure 2 and Figure S2.

**Figure 3:**
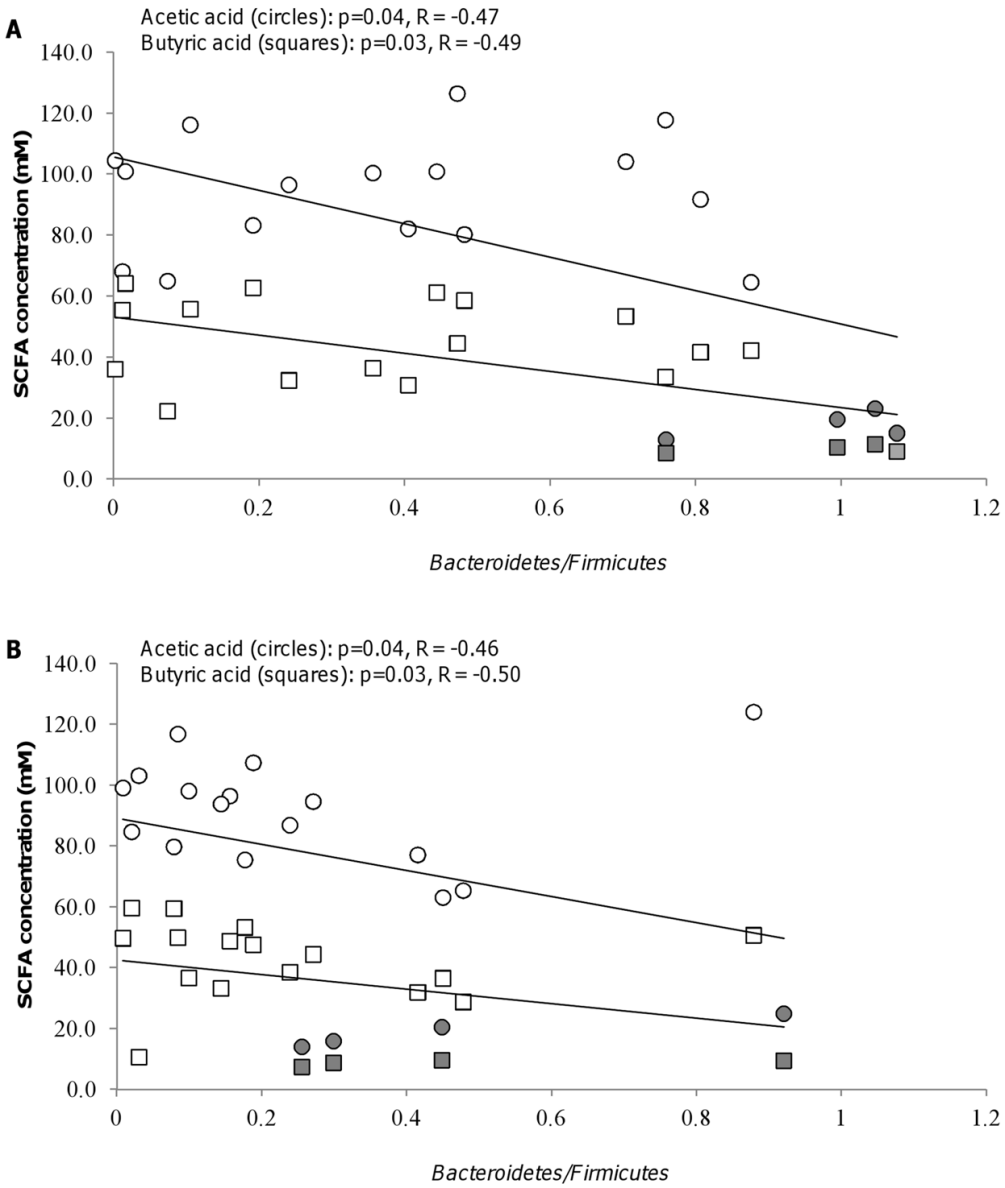
Correlation between Bacteroidetes/Firmicutes ratios and concentrations of SCFA. Correlation between concentrations of acetic acid (○) and butyric acid (□) and *Bacteroidetes/Firmicutes* ratios (*Bacteroides-Prevotella-Porphyromonas* group/*Clostridium perfringens* cluster I, *Clostridium coccoides - Eubacterium rectale* group and Clostridial cluster XIV) for synbiotic combinations with *Lactobacillus acidophilus* NCFM (A) and *Bifidobacterium animalis* subsp. *lactis* Bl-04 (B). Concentrations of acetic and butyric acid in control fermentations are denoted by grey circles and squares, respectively. The Spearman Rank probabilities (p) and correlations (R) are shown in the graphs.

All combinations increased the amount of both acetic and butyric acid by three to eight times as compared to levels in the control fermentations (Figure 2A and 2C) while levels of propionic acid were less increased (Figure 2B). For synbiotic fermentations, concentrations of acetic and butyric acid showed a tendency to decrease in vessels V3 and V4 as compared to V1 and V2, corresponding to the decreased saccharolytic activity reported from the ascending and transverse to the descending and sigmoid regions of the human colon [2]. Interestingly, there was a negative correlation between the modified ratio of *Bacteroidetes*/*Firmicutes* and concentrations of acetic and butyric acids, respectively, for both NCFM and Bl-04 synbiotics as seen from Figures 3A and 3B, respectively. No correlation of modified *Bacteroidetes*/*Firmicutes* ratio and concentration of propionic acid was observed.

In the NCFM fermentations, the stimulation of acetic acid production was strongest for isomaltulose followed by raffinose, OBGH and cellobiose, whereas in the Bl-04 fermentations the strongest induction of acetic acid was observed with raffinose, followed by xylobiose, melibiose and maltotriose. The strongest butyric acid inducer in the NCFM fermentations was raffinose followed by cellobiose, isomaltulose and OBGH, and in the Bl-04 fermentations melibiose followed by xylobiose, raffinose and maltotriose. Propionic acid production in NCFM fermentations was stimulated by all combinations except for NCFM in combination with raffinose (Figure 2B). For fermentations with Bl-04 synbiotics, all combinations but Bl-04 in combination with maltotriose showed a tendency of increasing levels of propionic acid.

No major alterations between the synbiotic treatments with regard to total levels of SCFA produced were observed. Total SCFA levels ranged between 532.9±71.2 and 693.2±35.2 mM for NCFM fermentations, and 553.3±57.4 and 631.4±57.9 mM for Bl-04 fermentations, demonstrating a four to five fold increase compared to SCFA in control fermentations (results not shown).

Significant reductions in concentrations of 2-methylbutyric, isovaleric and isobutyric acids, products of protein fermentation [57] were observed for all synbiotic combinations as compared to the controls (Figure S2) and no differences were observed between treatments. Concentrations of BCFA were lowest in vessel V1 and tended to rise throughout vessels V2 to V4 matching the increased proteolytic activity observed from the ascending colon toward the sigmoid colon [57].

## Discussion

In the past decades there has been increasing focus on the key role the gut microbiota appears to play in host health and disease. Further, probiotics, prebiotics and synbiotics can be used to modulate both composition and activity of the gut microbiota in a way beneficial to the human host [2], [16]. Traditionally increases in lactobacilli and bifidobacteria have been seen as particularly beneficial [29]. Recently, the ratios between *Bacteroidetes* and *Firmicutes* has become of special interest as it may be linked to so-called life style diseases such as obesity and type 2 diabetes [31], [33].

An important activity of the gut microbiota is formation of short-chain fatty acids (SCFA) [2]. SCFA contribute to human health by acting as an energy source for intestinal epithelial cells, and especially butyric acid has received much attention with reports indicating a range of functions ranging from anticarcinogenic to anti-inflammatory effects [38]–[40]. SCFA are able to inhibit pathogenic growth by lowering pH in the intestinal lumen [2] and acetic acid, produced by bifidobacteria, has been reported to improve defense of epithelial cells towards infection by pathogenic *Escherichia coli* O157:H7 [41]. For synbiotics, studies of their effects on the intestinal microbiota and its metabolic activity are limited and so far, rarely based upon combinations of well studied probiotics and prebiotics screened for actually stimulating growth of the particular probiotics as is the case in the present study.

For selection of growth promoting properties, a library consisting of 37 carbohydrates, with a wide range of different degrees of polymerization, glycosidic linkages and monomeric structures, was screened under laboratory conditions for their ability to stimulate growth of the well studied probiotic bacteria *Lactobacillus acidophilus* NCFM and *Bifidobacterium animalis* subsp. *lactis* Bl-04 [23], [51]–[55]. As expected, clear differences were observed between the carbohydrates. Further, the results are in line with previous studies with regard to stimulation of growth of Bl-04 and not NCFM by xylobiose and the lack of growth stimulation of both NCFM and Bl-04 by pullulan. But results differ from earlier findings in relation to growth of NCFM on polydextrose or panose and Bl-04 on gentiobiose [58]. The highest growth stimulation for both NCFM and Bl-04 was observed for carbohydrates with DP of two to five. To our knowledge, only the potential of xylobiose, gentiobiose, panose and pullulan in stimulating growth of NCFM and Bl-04 has previously been reported [46], [58]. The prebiotic potential of the remaining carbohydrates from the present study are, to our knowledge, for the first time investigated with respect to growth stimulation of NCFM and Bl-04.

The carbohydrates stimulating growth under laboratory conditions also were able to stimulate growth of the two probiotics in the colonic model system. Also oat β-glucan hydrolyzed by endo-1,3-β-D-glucanase (OBGH), which did not support growth of NCFM under laboratory conditions, was able to support growth of NCFM in the colonic model. The effect of OBGH in the simulated colonic conditions demonstrates the limitations of pure culture screenings, and suggests that in the presence of complex microbiota, the prebiotic effects of more complex carbohydrates are possibly enhanced, as compared to pure culture fermentations. It was observed, for all selected carbohydrates, that levels of NCFM and Bl-04 in the colonic model were higher than those of total lactobacilli and bifidobacteria, respectively. However, this was not observed in control fermentations and is most likely explained by a PCR bias caused by a high ratio of NCFM and Bl-04 as compared to other lactobacilli and bifidobacteria. Increase in cell numbers for a probiotic in the gastrointestinal tract is seen as a competitive advantage. In this regard the strongest effect of the carbohydrates tested was seen for NCFM; the levels of this probiotic were increased by 10^2^ to 10^4^ fold by all carbohydrates selected i.e. isomaltulose, cellobiose, raffinose and OBGH (Table 1A). The potential competitive advantage offered to Bl-04 by the carbohydrates tested was of smaller magnitude, amounting to 10 to 10^2^fold increases in the Bl-04 numbers. The results obtained indicate that specific prebiotics can be selected to provide a competitive advantage for a probiotic *Lactobacillus* species, probiotic *Bifidobacterium* species or both in a colonic model system.

Stimulation of SCFA by synbiotics including any of the candidate prebiotics used in the present study is to our knowledge only reported for raffinose in rats [59]. In *in vitro* studies only comprising prebiotics, i.e. melibiose, isomaltulose, cellobiose, xylobiose, maltotriose, raffinose and oat β-glucans, increases in acetic acid were reported to be between two and six times higher as compared to controls and for butyric acid the highest concentrations observed were four times higher as compared to control [60]–[64]. These increases in concentrations of acetic and butyric acids are, however, lower than the increases observed for all synbiotic combinations investigated in the present study where concentrations were three to eight times higher for both acetic and butyric acids as compared to control, The findings emphasize that a synergistic effect may be obtained when combining these prebiotic candidates with the probiotic strains NCFM and Bl-04.

NCFM is a homofermentative *Lactobacillus* strain which does not produce acetic acid and presence of NCFM alone does not increase production of acetic acid by the microbiota of this colonic model system [50]. Bifidobacteria produce acetic acid and increases of acetic acid in the colonic model are reported for another probiotic, *B. animalis* subsp. *lactis* Bi-07 [65]. Interestingly, the present study found that combinations of NCFM with carbohydrate stimulated production of acetic acid to the same extent as observed for Bl-04 in combination with carbohydrates (Figure 2A). We know of no previous studies reporting this and the present observation indicates that synbiotic combinations with NCFM and synbiotic combinations with Bl-04 may induce the same shift in metabolic activity of the microbiota and thereby potentially have the same SCFA mediated health benefits.

A shift in the modified ratio of *Bacteroidetes*/*Firmicutes* was seen in the presence of the eight different synbiotic combinations selected (Figure 1). To our knowledge the combined effect of probiotics and prebiotics on the ratio of *Bacteroidetes*/*Firmicutes* has not been previously reported. Regarding the effect of probiotics on the *Bacteroidetes/Firmicutes* ratio, only one published study seems to exist. This study, on children with atopic dermatitis and healthy controls, did not report effects for NCFM or *B. animalis* subsp. *lactis* Bi-07 on the ratio of *Bacteroidetes/Firmicutes* [52]. We are not aware of any previous studies reporting that prebiotics induce a shift in this ratio. The decrease in the modified ratio of *Bacteroidetes/Firmicutes* observed in the present study, was moreover found to be correlated to increases in concentrations of both acetic and butyric acid (Figure 3). Although shifts in *Bacteroidetes/Firmicutes* ratios have previously been reported to be associated with changes in acetic and butyric acids [32], direct correlations have to our knowledge not been reported previously. In the present study the donors were healthy lean subjects, and the findings cannot directly be compared to human studies. However, the ratios of modifed *Bacteroidetes/Firmicutes* observed in the presence of the selected synbiotic combinations varied between 0.002 and 0.9 (0.3–1.1 for control fermentations), and this variation is within the range of what has previously been reported for healthy humans, humans with diabetes type 2 and obese humans [31]–[33]. Although no conclusive link between the ratio of *Bacteroidetes/Firmicutes* and health status has been established, our results indicate that the synbiotic combinations investigated in the present study may be able to manipulate the composition of the microbiota, i.e. the modified ratio of *Bacteroidetes/Firmicutes,* in a way which could be important to human health.

Several potential prebiotics capable of stimulating the growth of NCFM and Bl-04 under laboratory conditions were identified, and the most interesting combinations were, in combination with the two probiotic strains, selected for further analysis in a human colonic model system. The selected combinations showed potential as synbiotics as they were able to support growth of the probiotic bacteria, affect the microbial composition, observed by a shift in the modified ratio of *Bacteroidetes/Firmicutes,* and shift the metabolic activity levels of the microbiota, demonstrated by an increase in concentrations of SCFA. The effects of the synbiotics on composition and activity of the microbiota remain to be confirmed by human trials.

## Materials and Methods

### Screening of Carbohydrates for Stimulation of Growth of *L. acidophilus* NCFM and *B. animalis* subsp. *lactis* Bl-04

Carbohydrates used in screening experiments are listed in Table S1. For the non-commercial carbohydrates prepared for this study (no. 20, 23–30), the degree of polymerization (DP) was determined by High Performance Anion Exchange Chromatography (HPAEC) chain profiling [66], except for oat β-glucan for which size exclusion chromatography was applied [67]. The library included carbohydrates with a large range of DP and a number of different monomeric units and glycosidic linkages (Table 2).

**Table 2:** Primers, mastermixes, standard al strains and annealing temperatures used in quantitative PCR detection of target bacteria.

Target bacteria	Primer	Standard bacterium	Annealingtemp. (°C)	Reference
*Lactobacillus acidophilus* NCFM	NCFM_F NCFM_R NCFMprobe	*Lactobacillus acidophilus* NCFM	61	[53], [55] (modified)
*Bifidobacterium animalis* subsp.*lactis*	Blact_1 Blact5	*Bifidobacterium animalis* subsp.*lactis* (HN019)	65	[64]; [72]
*Lactobacillus* spp.	Lab-0677 Lac1	*Lactobacillus acidophilus*(ATCC 43121)	56	[73]; [74]
*Bifidobacterium* spp.	BF BGR Bprobe	*Bifidobacterium adolescentis* (DSM20083)	60	[49]
*Bacteroides-Prevotella-* *Porphyromonas* group	gBacter_F gBacter_R	*Bacteroides fragilis* (ATCC 25285)	64	[75]
*Clostridium perfringes* cluster I	g_Cperf_F g_Cperf_R	*Clostridium perfringes* (ATCC13124)	55	[75]
*Clostridium coccoides -* *Eubacterium. rectale* group	g_Ccoc_F g_Ccoc_R	*Rumincoccus productus* (DSM 2050	61	[75]
Clostridial cluster XIV	CXIV F1 CXIV R2	*Clostridium bolteae* (DSM 15670)	52	[76]
*Faecalibacterium prausnitzii*	Fpraus_F Fpraus_R	*Faecalibacterium prausnitzii* (ATCC 27768)	62	[75]
*Enterobacteriaceae*	En-lsu3F En-lsu3’R	*Escherichia coli* (11775)	62	[77]

Screening was performed essentially as described previously [58]. Briefly, cultures of *L. acidophilus* NCFM and *B. animalis* subsp. *lactis* Bl-04 were pre-cultivated from stocks stored at −70°C, anaerobically for 24 h at 37°C in MRS broth (Lab M, Bury, United Kingdom). Anaerobic conditions were generated by the Hungate boiling system [68]. Modified MRS (without glucose) containing 1% (w/v) carbohydrate was inoculated with cell-suspensions of NCFM and Bl-04 (1% v/v). Modified MRS with no carbohydrate was used as control. Growth for 24 h at 37°C was monitored by optical density at 600 nm using a Bioscreen® C instrument (Labsystems, Helsinki, Finland) placed inside an anaerobic hood (80% N_2_, 10% CO_2_ and 10% H_2_). The area under the growth curves during 24 h was used to quantify growth [58]. Determinations were performed in two separate sets of experiments each in quadruplicate. Polydextrose (PDX;Danisco Sweetners, Redhill, UK), previously shown to have prebiotic effects [3], [49], [58] and glucose (Serva, Germany) were included for comparison.

### Four-stage Model of the Human Colon

Fermentations were performed in a four-stage semi-continuous model of the human colon (EnteroMix®, Danisco,) [49]. Shortly, the colonic model consists of four parallel units, each unit consisting of four vessels (V1–V4), connected sequentially and representing the different parts of the colon; ascending- (V1), transverse- (V2), descending colon (V3) and the sigmoid/rectum area (V4), respectively. Volumes were 6, 8, 10 and 12 mL, respectively, and pH was set at 5.5, 6.0, 6.5 and 7.0, respectively, and adjusted using gaseous ammonia in oxygen-free N_2_ gas. Fermentations were performed under thermostatic conditions (37°C). Faecal samples were obtained with verbal consent from healthy human volunteers (n = 3) and samples were preconditioned and incubated 24 hrs before use as previously described [49]. Faeces from one volunteer was used to run a set of four parallel fermentations. As faecal samples were anonymous and no medical or register was kept for the donors, there is according to national regulations no need for approval from the ethics committee nor is written consent demanded.

Candidate prebiotics were added (2% w/v) to synthetic ileal fluid [49], [69], used as basic medium, together with NCFM (lyophilized) or Bl-04 (oxygen-free 0.9% NaCl, as described above) at a rate of 2×10^7^ cells/mL (determined by flow cytometry (FACS Calibur-system; BD Biosciences, San Jose, CA, USA; as described by [70]). For control fermentations neither candidate prebiotic nor probiotic bacteria were added to the medium and fermentations were performed in quadruples, with one unit of the colonic model for the control and the remaining units for the synbiotic treatments. Fermentations were performed as independent duplicate runs for the synbiotic combinations whilst controls and the combination of Bl-04 and maltotriose were done in triplicate.

Operation of the model system was done as described previously [49] and fermentations were carried out for 48 h after which the content of each vessel was collected for further analysis. Bacteria from each vessel were harvested by centrifugation (48,000×g, 15 min, 20°C). The harvested bacteria and supernatant were stored at −70°C until DNA extraction and volatile fatty acid analysis.

### Determination of Microbial Numbers by Quantitative Real-time Polymerase Chain Reaction (qPCR)

DNA was extracted and purified (QIAamp® DNA Stool Mini Kit, Qiagen, Germany) according to manufacturer’s instructions with the following exceptions: an additional step of bead beating (45 s, 6.0 m/s in FastPrep ® FP120 instrument, Bio101 Savant Instruments, Inc. Holbrook, NY) with 1 g 1,000 µm glass beads (Sigma-Aldrich) was included prior to extraction, and lysis of bacterial cells was performed at 95°C instead of 70°C for 10 min.

Using specific primers and probes, and Taqman® or SYBR green methodology (Applied Biosystems, Warrington, USA) total densities of different bacteria were determined by qPCR. Target groups, methodology, annealing temperature, bacteria for standard curves and references are listed in Table 2. Assays were performed with ABI Prism® 7000 or 7500 FAST sequence Detection System (Applied Biosystems). Quantification was done using standard curves made by 10 fold dilutions series of target species DNA.

### Volatile Fatty Acid Analysis

Concentrations of short-chain fatty acids (SCFA), acetic, propionic, lactic and butyric acids, and branched-chain fatty acids (BCFA), 2-methylbutyric, isovaleric and isobutyric acid, using gas chromatography as described by Holben *et al*. (2002) [71].

### Statistical Analysis

The data of from the pure culture growth experiments are reported as the area under the growth curves, bacterial enumeration are expressed as log_10_ microbes/mL (±SE) and concentrations of volatile fatty acids in mM (±SE).

Data from control and synbiotic fermentations was compared by one-way ANOVA using Statistics Online Computational Resource (http://www.socr.ucla.edu/SOCR.html) and correlation between *Bacteroidetes/Firmicutes* and SCFA was computed by Spearman Rank correlation using Free Statistics Software (Office for Research and Development and Education, version 1.1.23-r7. http://www.wessa.net). P-values below 0.05 were considered significant.

## Supporting Information

Figure S1 **Growth of** ***Lactobacillus acidophilus*** **NCFM (dark grey) and** ***Bifidobacterium animalis*** **subsp.** ***lactis*** **Bl-04 (white) shown as area under the growth curve.** Carbohydrates are numbered 1–37 according to Table S1 and listed according to degree of polymerization (DP). The growth of Bl-04 on carbohydrates 4, 34 and 37 was not tested. Glucose and polydextrose (PDX) were included for comparison and results are shown as mean values ± standard error of mean (n = 8).
(TIF)

Figure S2
**Concentrations of branched-chain fatty acids as determined by gas chromatography; isobutyric acid (A), 2-methylbutyric acid (B) and isovaleric acid (C) in vessels V1–V4 of the colonic model after control and synbiotic fermentations.** Synbiotic fermentations are denoted as follows: *Lactobacillus acidophilus* NCFM in combination with; isomaltulose (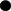)(n = 2), cellobiose (▴)(n = 2), raffinose (♦)(n = 2) and OBGH (▪)(n = 2) and *Bifidobacterium animalis* subsp. *lactis* Bl-04 in combination with; melibiose (○)(n = 2), xylobiose (Δ) (n = 2), raffinose (◊)(n = 2) and maltotriose (□)(n = 3). Control fermentations (n = 3) are denoted by crosses and dotted lines and results are shown as mean concentrations (mmol/L) for each vessel ± standard error of mean. *p<0.05, **p<0.005(TIF)

Table S1 **Carbohydrates screened for growth stimulation of** ***Lactobacillus acidophilus*** **NCFM and** ***Bifidobacterium animalis*** **subsp.** ***lactis*** **Bl-04 listed with names and manufacturers.** Size is given as degree of polymerization (DP).
(DOC)
