# Supplementary figures and images for: The Effect of Selected Synbiotics on Microbial Composition and Short-Chain Fatty Acid Production in a Model System of the Human Colon
Source: PLoS One. 2012 Oct 17;7(10):e47212. doi: 10.1371/journal.pone.0047212 (PMC3474826; doi:10.1371/journal.pone.0047212)

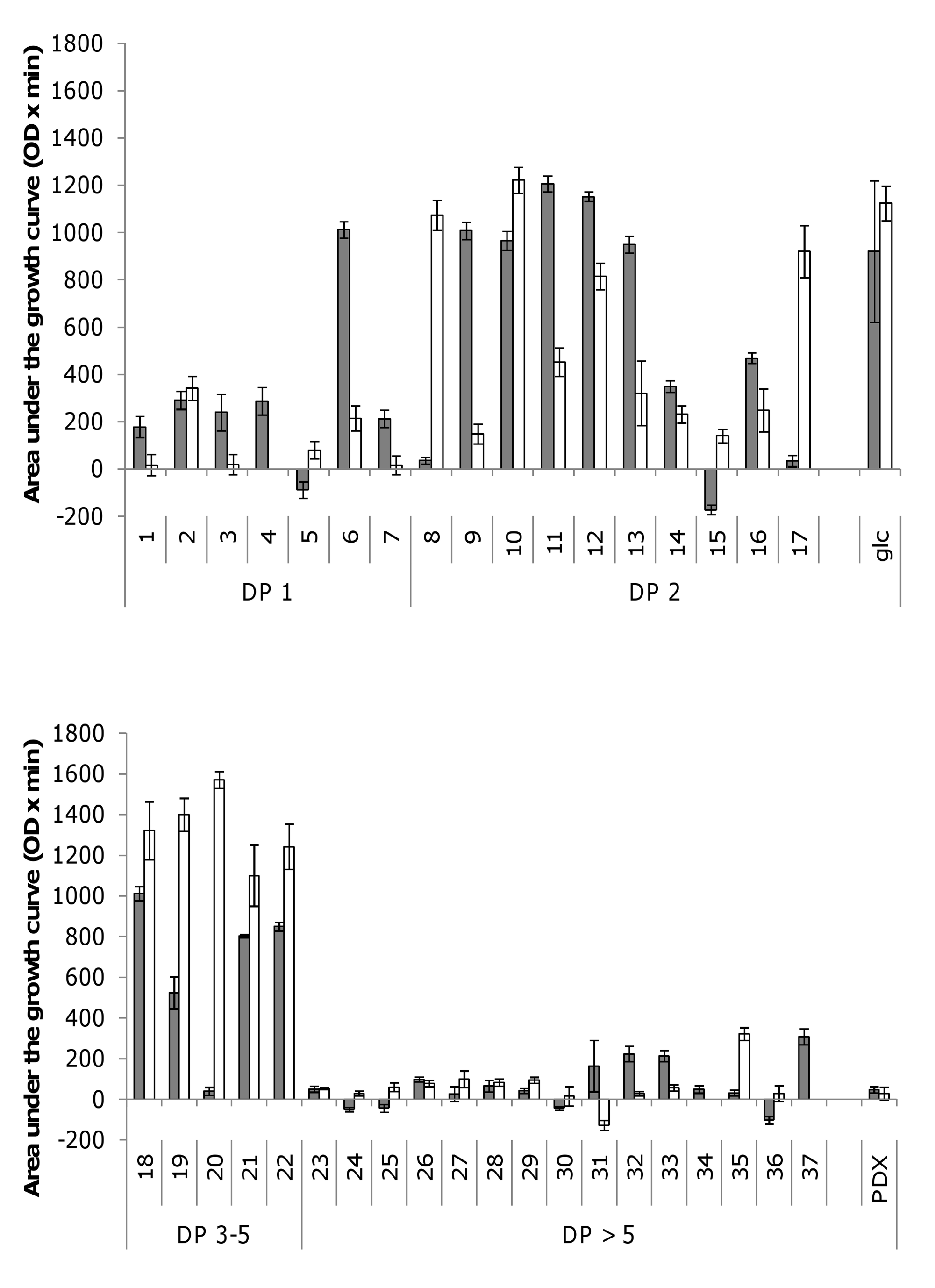

Supplement: Figure S1 — Growth of Lactobacillus acidophilus NCFM (dark grey) and Bifidobacterium animalis subsp. lactis Bl-04 (white) shown as area under the growth curve. Carbohydrates are numbered 1–37 according to Table S1 and listed according to degree of polymerization (DP). The growth of Bl-04 on carbohydrates 4, 34 and 37 was not tested. Glucose and polydextrose (PDX) were included for comparison and results are shown as mean values ± standard error of mean (n = 8). (TIF) [file pone.0047212.s001.tif]

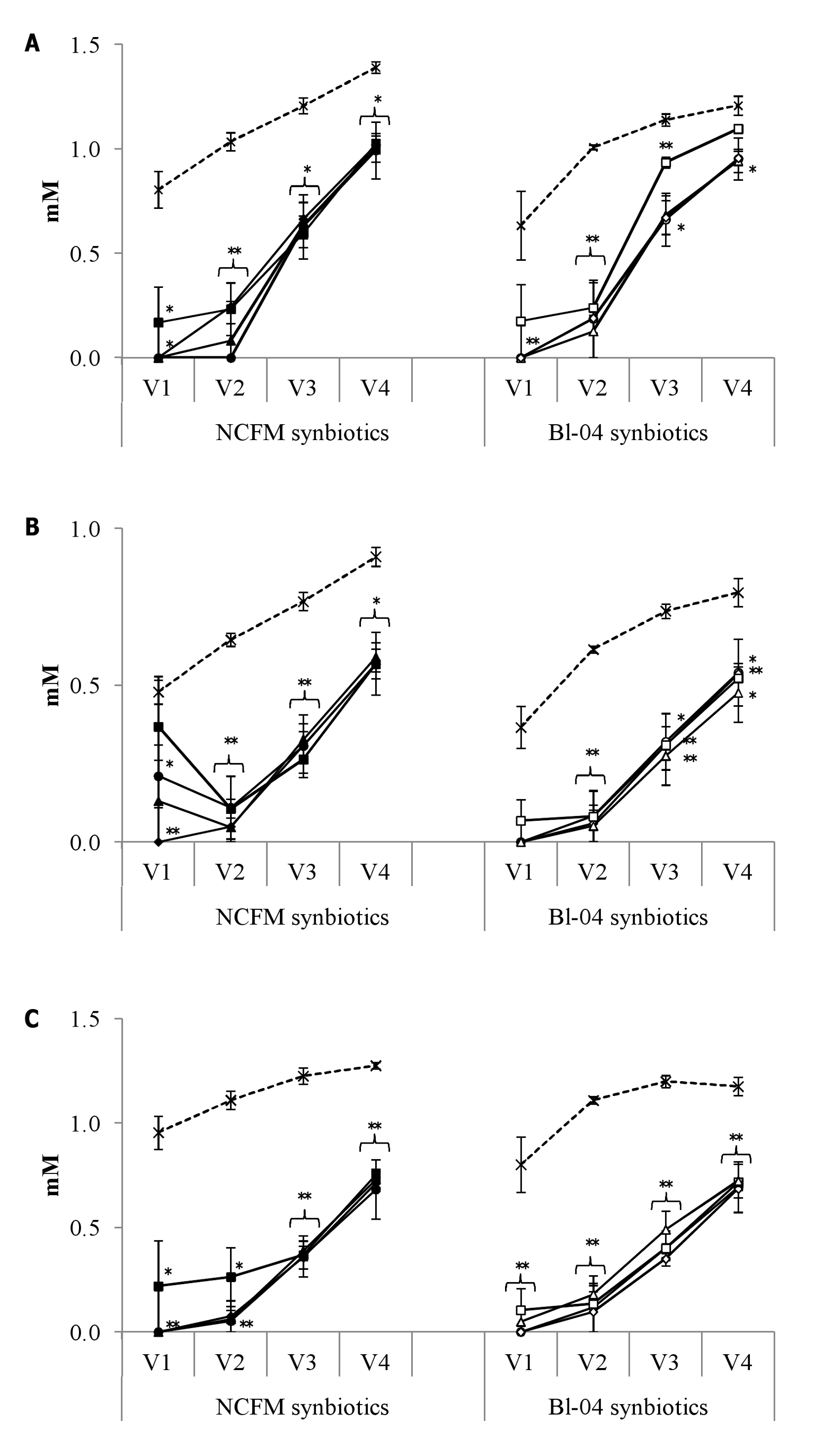

Supplement: Figure S2 — Concentrations of branched-chain fatty acids as determined by gas chromatography; isobutyric acid (A), 2-methylbutyric acid (B) and isovaleric acid (C) in vessels V1–V4 of the colonic model after control and synbiotic fermentations. Synbiotic fermentations are denoted as follows: Lactobacillus acidophilus NCFM in combination with; isomaltulose ()(n = 2), cellobiose (▴)(n = 2), raffinose (♦)(n = 2) and OBGH (▪)(n = 2) and Bifidobacterium animalis subsp. lactis Bl-04 in combination with; melibiose (○)(n = 2), xylobiose (Δ) (n = 2), raffinose (◊)(n = 2) and maltotriose (□)(n = 3). Control fermentations (n = 3) are denoted by crosses and dotted lines and results are shown as mean concentrations (mmol/L) for each vessel ± standard error of mean. *p<0.05, **p<0.005 (TIF) [file pone.0047212.s002.tif]
