# Supplementary material for: The Effect of Selected Synbiotics on Microbial Composition and Short-Chain Fatty Acid Production in a Model System of the Human Colon
Source: PLoS One. 2012 Oct 17;7(10):e47212. doi: 10.1371/journal.pone.0047212 (PMC3474826; doi:10.1371/journal.pone.0047212)
Supplement: Table S1 — Carbohydrates screened for growth stimulation of Lactobacillus acidophilus NCFM and Bifidobacterium animalis subsp. lactis Bl-04 listed with names and manufacturers. Size is given as degree of polymerization (DP). (DOC) [file pone.0047212.s003.doc]

Table S1. Carbohydrates screened for growth stimulation of *Lactobacillus* *acidophilus* NCFM and *Bifidobacterium* *animalis* subsp. *lactis* Bl-04 listed with names and manufacturers. Size is given as degree of polymerization (DP).

| **No.** | **Carbohydrate** | **Size** | **Manufacturer** |
| --- | --- | --- | --- |
| 1 | L-sorbose | DP 1 | Sigma-Aldrich |
| 2 | sorbitol | DP 1 | Fluka AG |
| 3 | xylitol | DP 1 | Sigma-Aldrich |
| 4 | tagatose | DP 1 | Sigma-Aldrich |
| 5 | D-mannose | DP 1 | Sigma-Aldrich |
| 6 | galactose | DP 1 | Sigma-Aldrich |
| 7 | xylose | DP 1 | Sigma-Aldrich |
| 8 | D-melibiose | DP 2 | Sigma-Aldrich |
| 9 | isomaltulose | DP 2 | Sigma-Aldrich |
| 10 | isomaltose | DP 2 | Sigma-Aldrich |
| 11 | cellobiose | DP 2 | Fluka AG |
| 12 | gentiobiose | DP 2 | Sigma-Aldrich |
| 13 | trehalose | DP 2 | Sigma-Aldrich |
| 14 | maltulose | DP 2 | Sigma-Aldrich |
| 15 | kojibiose | DP 2 | Wako Pure Chemicals, Osaka, Japan |
| 16 | nigerose | DP 2 | Wako Chemicals |
| 17 | xylobiose | DP 2 | Wako Chemicals |
| 18 | D-raffinose | DP 3 | Sigma-Aldrich |
| 19 | panose | DP 3 | Sigma-Aldrich |
| 20 | maltotriose (pullulan hydrolysate) ^a^ | mainly DP 3 | This study |
| 21 | stachyose | DP 4 | Sigma-Aldrich |
| 22 | verbascose | DP 5 | Megazyme, Wicklow, Ireland |
| 23 | β-glucan hydrolysate 1 ^b^ | DP >5 (up to DP 100) | This study |
| 24 | β-glucan hydrolysate 2 ^c^ | DP >5 (up to DP 100) | This study |
| 25 | β-glucan hydrolysate 3 ^d^ | DP >5 (up to DP 100) | This study |
| 26 | β-glucan hydrolysate 4 ^e^ | DP >5 (up to DP 100) | This study |
| 27 | galactan (lupin) hydrolysate ^f^ | DP >5 (up to DP 100) | This study |
| 28 | galactan (potato) hydrolysate ^f^ | DP >5 (up to DP 100) | This study |
| 29 | glucomannan (konjac) hydrolysate ^g^ | DP >5 (up to DP 100) | This study |
| 30 | galactomannan (carob) hydrolysate ^h^ | DP >5 (up to DP 100) | This study |
| 31 | alpha-cyclodextrin | DP 6 | Sigma-Aldrich |
| 32 | beta-cyclodextrin | DP 7 | Sigma-Aldrich |
| 33 | gamma-cyclodextrin | DP 8 | Merck |
| 34 | arabinoxylan wheat | DP >10 | Megazyme |
| 35 | pullulan | DP >10 | Megazyme |
| 36 | pea starch hydrolysate (batch 53) | average DP 22 | [1] |
| 37 | β glucan (oat) | average DP 50 | This study |

Hydrolysates were prepared as follows:

^a^ Pullulan fragments: pullulanase (Megazyme)*,* 2 units per 100mg pullulan at pH 4.5, 40°C for 150 min.

^b,c^ β-glucan fragments: endo-β-1,4-glucanase (EC 3.2.1.4) ^b^ NS 22018 (Biovelop) and ^c^ NS 22029 (Biovelop) from *Humicola insolens,* 4.0 unit per 100 mg of β-glucan at at pH 4.5, 40°C for 150 min.

^d^ β-glucan fragments: lichenase (EC 3.2.1.73) from *Bacillus subtilis* 1.0 unit per 100 mg of β-glucan at pH 4.5, 65°C for 150 minutes

^e^ β-glucan fragments: endo-1,3-β-D-glucanase (EC 3.2.1.39), from *Trichoderma* sp., 0.5 units per 100 mg of β-glucan at pH 4.5, 65°C 150 min.

^f^ Lupin and potato galactan fragments (Megazyme): galactanase, EC 3.2.1.89 from *Aspergillus niger,* 0.5 units, pH 4.5, 50°C, 180 min and 0.94 units, pH 2.8, 50°C, 150 min, per 100mg, respectively.

^g^ glucomannan fragments (konjac, Megazyme): endo-beta-1,4-mannanase (EC .2.1.78) from *Aspergillus nidulans* (AN6427.2 as described by [2]) 49.4 units per 100 mg glucomannan (Megazyme), pH 6.0, 40°C, 85 min.

^h^ galactomannan fragments: endo-beta-1,4-mannanase (EC 3.2.1.78) from *Aspergillus nidulans* (AN3358.2 as described by [2]) 242,4 units per 100 mg galactomannan (carob, Megazyme), pH 6.0, 40°C, 85 min

## References

1. Hansen M, Blennow A, Pedersen S, Engelsen S (2009) Enzyme modification of starch with amylomaltase results in increasing gel melting point. Carbohydrate Polymers 78: 72–79.

2. Dilokpimol A, Hiroyuki N, Gotfredsen CH, Baumann MJ, Nakai N, et al. (2011) Recombinant production and characterisation of two related GH5 endo-*β*- 1,4-mannanases from *Aspergillus nidulans* FGSC A4 showing distinctly different transglycosylation capacity. BBA-Proteins and Proteomics 1814: 1720–1729.

******

*****

*****

*****

*****

******

******

*****

*****

******

******

*****

******

******

******

*****

*****

******

*****

*****

******

******

*****
